# The role of gender in the active attitude toward treatment and health among older patients in primary health care—self-assessed health status and sociodemographic factors as moderators

**DOI:** 10.1186/s12877-017-0677-z

**Published:** 2017-12-08

**Authors:** Joanna Chylińska, Magdalena Łazarewicz, Marta Rzadkiewicz, Mirosława Adamus, Mariusz Jaworski, Gørill Haugan, Monica Lillefjel, Geir A. Espnes, Dorota Włodarczyk

**Affiliations:** 1 Department of Medical Psychology, Medical University of Warsaw, Ul. Żwirki i Wigury 81, 02-091 Warsaw, Poland; 2 NTNU Center for Health Promotion Research, Department of Public Health and Nursing, Norwegian University of Science and Technology, Trondheim, Norway; 3 NTNU Center for Health Promotion Research, Department of Neuromedicine and Movement Science, Norwegian University of Science and Technology, Trondheim, Norway

**Keywords:** Ageing, Health promotion, Men’s health, Gender, Patient adherence

## Abstract

**Background:**

Active attitude toward treatment and health (ATH) leads to improved cooperation and better health outcomes in patients. Supporting it in the population of older adults is a growing need in primary care. Recognising the role of gender, health and other sociodemographic factors can help to distinguish patients who need the most assistance in activation from general practitioners (GPs). The objective of the study was to investigate gender differences in ATH as well as the moderating role of self-assessed health (SAH) and selected sociodemographic factors (age, education, financial status, marital status).

**Methods:**

A cross-sectional, multicentre study among 4936 primary care older patients (aged 50+) was conducted. The PRACTA-Attitude toward Treatment and Health questionnaire (PRACTA-ATH) was used to measure the cognitive, emotional (positive and negative affect), and motivational dimensions of ATH. Patients were approached before and after their visits in the primary health-care facilities randomly selected in Central Poland.

**Results:**

Generalised linear models (GENLIN) revealed the main effects of gender, SAH, and sociodemographic characteristics, such as financial status, marital status and education. Interaction effects of gender and age (Wald’s χ^2^ = 24.767, *p* < 0.001 for ATH Global), as well as gender and SAH (Wald’s χ^2^ = 16.712, *p* < 0.002 for ATH Global) on ATH were found. The most assistance in regard to ATH was required by men aged 50–74 and men declaring good self-assessed health. Generally, women declared a more active attitude than men, showing more knowledge (M = 5.40, SD = 0.07 and M = 5.21, SD = 0.07, for women and men, respectively, *p* = 0.046), positive emotion (M = 5.55, SD = 0.06 and M = 5.33, SD =0.06, for women and men, respectively, *p* = 0.015) and motivation to be involved in their health issues (M = 5.71, SD = 0.07 and M = 5.39, SD = 0.07, for women and men, respectively, *p* = 0.001). The level of negative emotions related to health was not significantly different between genders (*p* = 0.971).

**Conclusions:**

The need to create health promoting programmes taking account of particular gender differences in older adults emerges. In regard to clinical practice, building a sense of efficacy and individual responsibility for health, providing information about the means of health promotion and prevention, and recognising health-related cognitions, is recommended especially for men who feel well and are less advanced in age (50–74).

**Electronic supplementary material:**

The online version of this article (10.1186/s12877-017-0677-z) contains supplementary material, which is available to authorized users.

## Background

As a result of demographic changes, the group of older patients is growing in primary care as their health status and functional ability deteriorate with time. The World Health Organization points out that fostering good health and improving the quality of life in older adults shall be of central interest, because these goals contribute to better productivity and greater independence [1]. Patients who actively take care of their health, and who feel responsible and in control of their medical care, achieve better health outcomes [2–4]. General practice is one of the specialties in which facilitation of an active attitude toward health (ATH) is expected by the patients themselves [5, 6].

Research into active ATH typically refers to the unidimensional approach, in which patients are assigned to one of the categories ranging from passive to active. However, the multidimensional concept of ATH, which encompasses three dimensions, cognitive, emotional, and motivational, may offer health providers more insight into the process of patient activation. Unlike non-modifiable factors, such as age, financial status, or level of education, ATH is subject to change and can be easily facilitated by health providers in a clinical setting. Three components may answer the question about what specific aspect of the attitude needs to be changed. The cognitive aspect covers the area of patients’ health beliefs and illness cognitions, as well as expectations of treatment outcomes and medical care. The emotional component encompasses both the positive and negative affective states experienced with health issues. The motivational component includes patients’ intentions, plans, and actual behaviors related to their health [7]. Subsequently, comparing gender on several dimensions of ATH instead of only one may provide information about particular informational, emotional, and motivational needs of older adults, which can be addressed to increase the level of activation. GPs can evaluate more accurately which gender needs more health education, emotional support, or motivation building, beyond simply knowing the general level of a patient’s engagement, which is provided by the unidimensional approach.

The literature suggests that patients who are able to understand their health problems and hold realistic views of their state of health, and who respond with adequate emotions and are motivated to cooperate with clinicians, achieve favorable health outcomes [8]. The traditional three-dimensional model of the attitude is well grounded in psychological theory and posits beliefs/cognitions, emotions, and behavior as mutually interrelated and affecting each other [9–11]. The interrelation of the three components has also been acknowledged empirically in the area of health: Perceptions of illness or cognitive representations directly influence an individual’s emotional response, which in turn determines how patients respond to the health condition in their coping behavior, such as adherence to treatment [12–14].

Data on gender-related differences in patients’ multidimensional active attitudes are not directly available, because, to our knowledge, all three aspects included in ATH have not yet been studied together. However, when we consider each dimension separately, the data are contradictory. On one hand, when we consider cognitive and motivational aspects of ATH, it has been reported [15] that being female was a strong predictor of being proactive, well-informed, and involved in health issues; this conforms with existing research in the area of cognitive processing and management of health-related information. Also, in most research, men tend to engage in riskier health behaviors [16]. Gender differences in emotional aspects of ATH have not been investigated, but data on general mental well-being favors men [17, 18]. On the other hand, when a unidimensional construct of patient activation was studied, reports indicated more favorable results for men [19, 20] or no gender differences [21–23]. In an investigation of the relationship between ATH and gender, other factors can possibly play a role. In the present paper, we also examine self-rated health and a set of sociodemographic factors.

Health state is related to both gender and ATH. Detailed descriptions of gender-related disparities in this area are available in the literature. Women receive lower wages and, therefore, are more likely to have lower socio-economic status (SES). They also experience more stressful life events and chronic stressors. Each of these factors is related to health indices [24]. Differences between women and men in health are well established, suggesting significant differences in physical functioning, disability, and self-assessed health measures: Men score better on these indicators. On the contrary, women live longer and experience fewer life-threatening medical conditions [24]. It is important to note that gender disparities regarding health status change with age. In late adulthood, women consistently report more functional limitations than men, while the gap between genders in self-assessed health closes [24, 25]. Also, gender-related differences in health-behavior patterns decrease with age [15]. Patient activation among older adults is positively correlated with health as reported by Smith et al. [26]. In this research, health was operationalized with the results on SF-36, which correlates only in part [27] with self-assessed health (SAH). Other sociodemographic factors, such as marital status and SES, have been investigated for patient activation [28, 29], but not for multidimensional ATH.

As presented above, existing data provide only some approximations of understanding the role of gender on each of the dimensions that ATH combines. Comprehensive understanding of a patient’s ATH allows a doctor to know if more emphasis on providing information, motivating, reducing negative emotions, or increasing positive ones should be invested in building a professional, patient-centered relationship.

## Methods

### Aim of the study

Understanding the relationship between ATH and gender, as well as clarifying the role of SAH, age, and selected sociodemographic factors, would allow health providers to understand older patients’ attitudes better. Facilitation of appropriate elements of ATH, congruent with gender, SAH, and demographic effects investigated in the research, may result in greater activation and better health outcomes. Therefore, the aim of the study was to detect gender differences in ATH in primary care among older adults. Because the study proposes a novel approach that encompasses cognitive, emotional, and motivational aspects of ATH, and because the available literature is contradictory, three research questions were investigated:

1. Are there gender differences in the ATH of older adults visiting primary health-care physicians?
2. Are sociodemographic factors and health related to the ATH of older patients?
3. Can the relationship between gender and ATH be moderated by sociodemographic factors and health?

### Participants and procedure

The study was part of a Polish–Norwegian research project aimed at activating the older adults in medical practices. The participants included 4936 patients attending primary health-care facilities (PHCF) in central Poland, funded by the National Health Fund. Simple randomization in selection of PHCS was performed, and a 20% response rate was obtained. The number of patients was related to the number of GPs participating in the project (detailed description in [30]). Ten consecutive patients of each GP recruited for the study were approached in the waiting room before their visit took place. If patients agreed to participate, they filled in a set of questionnaires before and after the visit, based on the design of the project. None of the tools administered before the visit could affect the results of PRACTA-ATH, which was completed after the visit (together with other questionnaires used in the PRACTA project).

The inclusion criteria were: being a patient assigned to the GP recruited for the project, waiting for a visit on a given day, above age 50, possessing cognitive abilities to be able to fill in a questionnaire independently, and consent to participate. Patient eligibility was screened by professional pollsters while collecting data. They had been trained in the procedure and inclusion criteria before beginning their work. Their decision about a patient’s cognitive competency was based on observations made during the process of inviting participants to take part in the study. The patient response rate was 76.6%.

### Measures

The ATH was evaluated with the PRACTA-Attitude toward Treatment and Health Scale (PRACTA-ATH; 16 items). PRACTA-ATH allowed four aspects of the attitude to be assessed: cognitive, emotional-positive, emotional-negative, and motivational. The cognitive aspect referred to the level of a patient’s understanding of disease and its treatment; the emotional component was split into two subscales measuring separately positive and negative emotions related to a patient’s health status, and the motivational component covered a patient’s intentions and plans for active participation in the treatment. Evaluation of ATH was performed in connection with the visit, after which patients were administered this scale They responded to items starting with the statement: Due to this visit to the doctor … (i.e. I understand the cause of my ailments, feel calmer, I think I can influence how I’ll feel in the future, etc.). Answers were given on a 7-point Likert scale (1 = definitely not, 7 = definitely yes). Higher scores suggest a more active attitude in all aspects, except negative emotions. The global score for the PRACTA-ATH was counted by creating a mean from all four dimensions (the results in negative emotions had to be reversed).

The internal consistency of the PRACTA-ATH was good with the Cronbach’s alpha = 0.88; the subscales’ internal consistency was good to excellent with the Cronbach’s alpha ranging from 0.88 to 0.93 (unpublished data, 2017).

Data regarding sociodemographic characteristics (age, gender, financial situation, marital status, and education) and comparative SAH were collected before the consultation, together with a set of other questionnaires not included in the present study. Participants provided information about marital status, choosing one of four available categories: single, marriage/partnership, divorced/separated, widowed. Education was reported in five categories (primary, vocational, secondary without matura exam, secondary with matura exam, higher); however, for the purpose of the analyses, three main groups were created (less than secondary, secondary, and higher), as a result of distribution and to increase the clarity of the results. Financial situation was measured by responses on a 5-point Likert scale (1 = poor, 5 = good) to the question: “How do you evaluate your financial situation?” SAH was operationalized as a single item (“How do you evaluate your health in comparison to people of similar age?”) with a response scale ranging from 1 (very good) to 5 (very poor).

### Statistical analyses

Statistical analyses were performed on IBM SPSS Statistics software, versions 23 and 24. The level of significance assumed in the study was *p* < 0.05. The Kolmogorov–Smirnov test with the Lillieforce correction was used to evaluate normality of distribution, and chi-square tests were applied to screen for gender differences in sociodemographic characteristics. To calculate the effect size of the detected differences, Cramér’s *V* was used, and a *Z*-test was applied to estimate the significance of differences between categories.

Owing to the non-normal distribution of outcome variables (*p* < 0.001), generalized linear models (GENLIN) were performed as an alternative to general linear models (distribution-gamma; link-identity) [31]. Gender, age, SAH, level of education, and marital and financial status were included as factors in the model. Additionally, interactions of gender with each of the factors were also included (in an order equivalent to the order of main factors). Separate models for the global score in ATH and for each of its dimensions were built and evaluated. The results of GENLIN procedures allowed us to verify all three research questions. The results of the Wald’s chi-square test calculated for each of the factors included in the model allowed us to answer the first and second research questions, while the results of the Wald’s chi-square tests for interaction effects were a base on which to answer the third research question. Pairwise comparisons performed within GENLIN were used to detect significant differences between given categories.

## Results

### Characteristics of the participants

The age of the participants ranged from 50 to 98 (*M* = 68.85, *SD* = 9.1). Four age groups were considered in the study: (1) patients aged 50–64 (31.1%), (2) patients aged 65–74 (40.9%), (3) patients aged 75–84 (24%), and (4) patients above 85 years old (4.1%). The study comprised 2864 women (58%) and 2072 men (42%). Preliminary analyses were aimed at discovering gender differences in independent variables. As presented in Table 1, women differed significantly from men in aspects such as age, health, marital status, and financial status. However, the effect size of obtained differences (Cramér’s *V* ranging from 0.04 to 0.11) were small. No differences were detected in the level of education. Significantly fewer women were under 65, and more were aged 75–84; also, fewer women described their health state as good, but more than men did, on average. More men remained married or divorced, and declared an average financial status, while more women declared themselves as poor or very poor and widowed (all given differences, statistically significant values of a *Z*-test).

**Table 1:** Gender differences in demographic variables (*n* = 4936)

	Category	Female		Male		Chi^2^(Kramer’s V) p
		(*n* = 2864)		(*n* = 2072)		
		N	%	N	%	
Age groups	50–65	847_a_	29.6	682_b_	33.1	10.05 (0.045)
	65–74	1176_a_	41.1	835_a_	40.5	*p* = 0.018
	75–84	723_a_	25.3	457_b_	22.2	
	85+	112_a_	3.9	89_a_	4.3	
Health	Very good	53_a_	1.9	44_a_	2.1	15.42 (0.056)
	Good	578_a_	20.2	512_b_	24.7	*p* = 0.004
	Average	1717_a_	60.0	1168_b_	56.4	
	Poor	468_a_	16.3	313_a_	15.1	
	Very poor	48_a_	1.7	35_a_	1.7	
Education	Less than secondary	1204_a_	42.0	846_a_	40.8	0.76 (0.012)
	Secondary	1200_a_	41.9	890_a_	43.0	*p* = 0.685
	Higher	460_a_	16.1	336_a_	16.2	
Marital status	Single	148_a_	5.2	101_a_	4.9	56.57(0.107)
	Marriage	1580_a_	55.2	1284_b_	62.0	p < 0.001
	Divorced	171_a_	6.0	180_b_	8.7	
	Widowed	965_a_	33.7	507_b_	24.5	
Financial status	Poor	89_a_	3.1	43_b_	2.1	13.74 (0.053)
	Rather poor	533_a_	18.6	339_b_	16.4	*p* = 0.008
	Average	1603_a_	56.0	1232_a_	59.6	
	Rather good	535_a_	18.7	365_a_	17.6	
	Good	104_a_	3.6	93_a_	4.5	

Lower indexes indicate the results of Z-test: every letter represents the subcategory of gender for which the column proportions do not differ significantly on the level of 0.05

### Main effects of gender, SAH, and sociodemographic factors on ATH

The main effects of all analyzed factors (such as gender, age, health, education, and marital and financial status) on ATH, as well as interactions of factors with gender, were investigated. In all models, the same number and order of factors were maintained. The same procedure was applied for each dependent variable: all of the aspects and the global score of the ATH. In the model for the cognitive component, one of the interaction factors (gender x financial situation) had to be removed from the model because the Hessian matrix was undefined. This decision was supported by the fact that this factor was insignificant when it was analyzed independently.

Results of the Wald’s chi-square test performed for Global PRACTA-ATH and each of its subscales are presented in Table 2.

**Table 2:** Omnibus test results for attitude toward treatment and health

Attitude toward health	Global		Cognitive		Positive emotions		Negative emotions		Motivation	
	Wald’s Chi^2^	p	Wald’s Chi^2^	p	Wald’s Chi^2^	p	Wald’s Chi^2^	p	Wald’s Chi^2^	p
Intercept	20,483.378	< 0.001	11,326.505	< 0.001	14,753.063	< 0.001	2248.749	< 0.001	12,213.085	< 0.001
Gender	**6.122**	**0.013**	**3.965**	**0.046**	**5.902**	**0.015**	0.001	0.971	**10.415**	**0.001**
Age	**24.771**	**< 0.001**	**53.191**	**< 0.001**	**28.885**	**< 0.001**	**43.180**	**< 0.001**	**38.547**	**< 0.001**
Health	**109.220**	**< 0.001**	**74.056**	**< 0.001**	**48.594**	**< 0.001**	**117.156**	**< 0.001**	**26.232**	**< 0.001**
Education	**14.418**	**0.001**	**11.200**	**0.004**	**8.064**	**0.018**	**7.374**	**0.025**	**11.310**	**0.004**
Marital status	**97.324**	**< 0.001**	**91.574**	**< 0.001**	**54.991**	**< 0.001**	3.862	0.277	**78.457**	**< 0.001**
Financial status	**135.238**	**< 0.001**	**80.235**	**< 0.001**	**120.381**	**< 0.001**	**33.804**	**< 0.001**	**111.074**	**< 0.001**
Gender x age	**24.769**	**< 0.001**	**19.341**	**< 0.001**	**27.759**	**< 0.001**	3.273	0.351	**31.993**	**< 0.001**
Gender x health	**16.712**	**0.002**	**16.516**	**0.002**	**22.104**	**< 0.001**	8.477	0.076	**29.361**	**< 0.001**
Gender x education	4.258	0.119	2.647	0.266	5.349	0.069	4.205	0.122	0.884	0.643
Gender x marital status	1.186	0.756	2.191	0.534	0.389	0.942	4.864	0.182	0.921	0.820
Gender x financial status	4.584	0.333	–	–	5.115	0.276	13.044	0.011	4.010	0.405

Statistically significant results are marked in bold

The findings indicated that similar factors were significant in all models: The main effects of gender, age, SAH, education, and marital status were found in all investigated dimensions, except for negative emotions, where neither gender nor marital-status effects were detected.

Across the subscales, gender interacted significantly with age and health, but not with education, marital status, or financial situation. Again, the negative-emotions subscale was an exception with no interactions at all. In the global score of ATH and health, both main and interaction effects of gender were obtained (Table 2).

#### Impact of gender on ATH

The results clearly indicate that gender is related to ATH, because both main and interactional effects were found. Generally, women evaluated their attitude as more active than men did, although compared with other variables, such as financial status, health, and marital status, the effects of gender were rather weak. For instance, in ATH Global, Wald’s chi-square test for gender = 6.12 (*p* = 0.013), while Wald’s chi-square test for financial status = 135.24 (*p* < 0.001) and for SAH = 109.22 (*p* < 0.001; see Table 2 for the remaining results). The results of pairwise comparisons (presented in Table 3) indicate that women reported a more active attitude, understand and know more about their health (*M* = 5.40, *SD* = 0.49 for women, *M* = 5.21, *SD* = 0.07 for men; Wald’s chi-square test = 3.96, *p* = 0.046), experience more positive emotions (*M* = 5.55, *SD* = 0.06 for women and *M* = 5.33, *SD* = 0.06 for men; Wald’s chi-square test = 5.90, *p* = 0.015), and have stronger motivation to be engaged in their health issues (*M* = 5.71, *SD* = 0.07 for women and *M* = 5.39, *SD* = 0.07 for men; Wald’s chi-square test = 10.41; *p* = 0.001). Gender differences were not found in the dimension of negative emotions only.

**Table 3:** Gender differences in ATH

Variable	Women		Men		p
	M	SD	M	SD	
ATH Global	5.39	0.49	5.21	0.05	0.013
Cognitive	5.40	0.07	5.21	0.07	0.046
Positive Emotions	5.55	0.06	5.33	0.06	0.015
Negative Emotions	3.22	0.09	3.23	0.10	0.971
Motivation	5.71	0.07	5.39	0.07	0.001

The main effects of gender, however, have to be interpreted with caution, because the interaction effects of gender provide further details about the investigated relationship.

#### The effects of the Sociodemographic factors and SAH on ATH

SAH, as well as age, education, and financial and marital situation, was also related to ATH (see Table 2 for results of the Wald’s chi-square test results). Pairwise comparisons for age groups revealed that the most active were patients aged 65–84, because groups of patients aged 65–74 and 75–84 (*M* = 5.40, *SD* = 0.03, and *M* = 5.34, *SD* = 0.04, respectively) did not differ significantly (*p* = 0.21). The oldest old (85+) and the youngest old (50–64) groups seem to be more passive (*M* = 5.26, *SD* = 0.03, and *M* = 5.19, *SD* = 0.07, respectively); these groups didn’t differ significantly from each other (*p* = 0.26), but significance was found in comparisons with the two remaining groups.

Comparisons of ATH Global means for SAH groups reveal that patients declaring better health (average and higher categories) scored higher (*M* = 5.35, *SD* = 0.09 for very good SAH; *M* = 5.41, *SD* = 0.04 for good SAH; and *M* = 5.46, *SD* = 0.03 for average SAH) than those who assessed their health as poor (*M* = 5.08, *SD* = 0.04) or very poor (*M* = 5.19, *SD* = 0.10). However, statistical significance was obtained for differences between the “poor” group and three more healthy groups only (*p* = 0.007, *p* < 0.001 and *p* < 0.001 for subsequent categories starting with the “very good SAH” group). Patients describing their health as very poor didn’t differ significantly from poor, and interestingly not from very good either. Standard error may be responsible for the lack of these differences.

Education level also affected ATH; the most active attitude was found in patients with the lowest educational level (*M* = 5.36, *SD* = 0.04). These patients differed significantly from participants with a secondary education (*M* = 5.25, *SD* = 0.04), but not from patients with a higher education (*M* = 5.29, *SD* = 0.04).

Marital and financial situations were also related to ATH. The most active group (differing significantly from all other groups) was that of divorced patients (*M* = 5.32, *SD* = 0.06), while the most passive was that of married individuals (*M* = 5.08, *SD* = 0.06), who differed significantly from all remaining groups as well. Analyses for financial status suggest a gradual increase in active attitude with improvement of the material situation. The groups declaring a poor (*M* = 5.03, *SD* = 0.05) or very poor (*M* = 5.04, *SD* = 0.08) financial status didn’t differ from each other (*p* = 0.89), but each of the subsequent groups scored significantly higher than a preceding one (*M* = 5.22, *SD* = 0.04 for average financial situation; *M* = 5.48, *SD* = 0.05 for good situation; and *M* = 5.72, *SD* = 0.07 for very good financial status).

### Sociodemographic factors and health as moderator of the ATH–gender relationship

To analyze the potentially moderating factors of the ATH–gender relationship, interaction effects of gender and sociodemographic factors as well as health were studied. Across the results, we found only two significant interaction factors: age and health. None of the remaining sociodemographic factors in the study moderated the investigated relationship (Table 2). Both interactions appeared as significant in models for the global score of ATH and its three dimensions: cognitive, positive emotions, and motivation. They were not found in the model for negative emotions (Table 2).

**Fig. 1:**
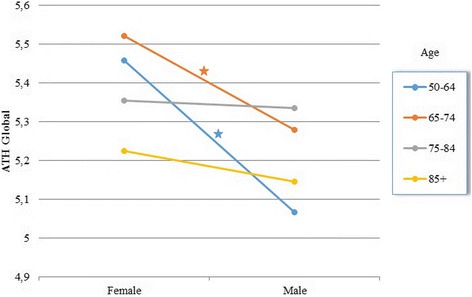
Interaction effect of age and gender on ATH Global

Pairwise comparisons performed for the ATH Global indicate that the interaction effect of gender and age reflects significant differences in the two younger groups of patients (aged 50–74; *M* = 5.45, *SD* = 0.05, and *M* = 5.07, *SD* = 0.06, *p* < 0.001 for women and men aged 50–64, respectively; *M* = 5.52, *SD* = 0.05, and *M* = 5.27, *SD* = 0.06; *p* = 0.001 for women and men aged 65–74, respectively), which disappear in participants aged 75 or more (*p* > 0.05; Fig. 1).

**Fig. 2:**
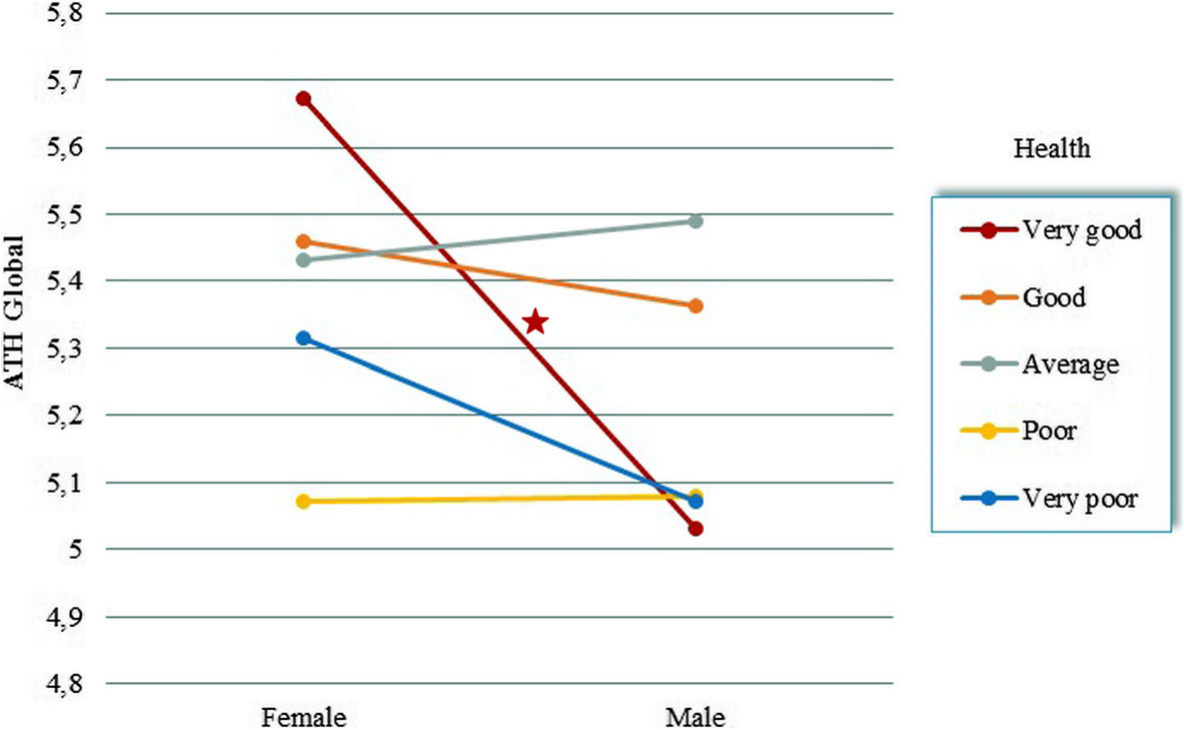
Interaction effect of SAH and gender on ATH Global

The difference responsible for the interaction effect of gender and health occurred in the group of patients with very good health. Women in this group rated their attitude significantly higher (*M* = 5.67; *SD* = 0.14) than men did (*M* = 5.03, *SD* = 0.13; *p* < 0.001). No significant gender differences were detected on remaining levels of health status (*p* > 0.05; Fig. 2).

Interactions of gender with age and health were systematically observed in all dimensions of the PRACTA-ATH scale, except for negative emotions. Pairwise comparisons repeated findings from ATH Global; women in older age groups (75 or older) resembled men in their knowledge, intensity of the positive affect, and motivation, while groups aged 50–74 differed significantly, with men having a less favorable attitude (for details, see Additional file 1: Table S1).

The consistency of results also remains for the type of differences observed between genders in the health groups. For positive emotions and motivation scales where the interactions of gender and health were found, significant gender differences exist only in the group declaring very good health. We observed an additional gender difference in the positive emotions subscale. Here, patients with very poor health also differed significantly in gender (*p* = 0.007). Again, women were able to have more positive emotions (*M* = 5.52, *SD* = 0.16) than men (*M* = 4.88, *SD* = 0.17).

## Discussion

In our study, we investigated if gender, SAH, and other sociodemographic factors could be related to the level of active ATH, understood as a multidimensional construct, in a group of older adults attending primary care.

In our investigation, the strongest gender differences were detected in groups of younger cohorts of older adults (aged 50–74), with women exhibiting more active ATH (the exception was the negative emotions scale, where no differences were found). In the literature, gender has been found to be related to patients’ activation [3, 4], but the direction of differences was not clear because contradictory results are available [15, 19]. In our study, men were more in need of interventions that would facilitate their active ATH. Gender differences disappeared among patients older than 75; this is in line with other research demonstrating that gaps between genders close with age [15, 24] and specifying that this pertains also to ATH.

Interestingly, the SAH-induced gender differences appear only in the group of patients describing their health as very good. Men from this group had the most passive attitude, while women declared the most active attitude of all groups, which suggests the need for an intervention aimed at raising men’s awareness and motivation to protect and sustain well-being despite the fact that they are feeling well.

Similar results were found across subscales of ATH except negative emotions. In the general population, women reveal higher rates of psychological symptoms (such as insomnia or nervousness) [32] and report greater fear in addition to having higher prevalence rates of anxiety disorders. Depression, one of the most urgent geriatric problems in mental health, affects older women more often. Contrary to results for general well-being regarding health-related issues, women tend to have more hope and are more optimistic than men. This result suggests that men should not be stereotyped as “being strong”. They require support to help them regulate their emotional distress.

Our results on gender shouldn’t be interpreted apart from other findings from the study, which point to the importance of other sociodemographic factors.

When age differences were considered, the most passive patients were either relatively young (50–64) or old (above 85). In both groups, attitudes toward health might be anchored in specific cognitions regarding health and health care, but the content of these beliefs would probably be different. Possibly, the first group may not be affected by health problems yet, and at the same time, may not fully understand the possibility of undertaking actions to sustain well-being; this results in lower interest and motivation in taking health-related actions. The latter has to face increasing limitations and decline related to age, which might increase patients’ sense of helplessness/hopelessness, and which subsequently lead to a less active ATH.

Also, patients who declared poor health or poor SES were less active. The most consistent findings in the literature, repeated in the present work, point to a positive relation between an active attitude and SES. Better self-assessed heath has also been found to coexist with more activation in patients [19]. This positive relationship remains true, regardless of the age of older patients. Older adults become most involved with their health when they are between 65 and 84; both earlier and later, they declare more passivity. In other studies, younger age results in more activation in both general [19] and older populations. Our results indicate that old adulthood has its dynamics, and age differences within a cohort of these patients are visible.

Marital status is another factor that was related to ATH. The most passive were married patients, who significantly differed from divorced ones. At first glance, it might seem a surprising result. On one hand, married patients would be expected to experience more social support, which should result in a more active attitude. Conversely, a systematic review of studies of chronic-illness self-management found that patients who lived alone attended to their health needs more actively because they had to manage their health independently and had no immediate help available. Also, they were found to value social support more and sought it outside the home [33].

The impact of education on ATH contradicts the data available in the literature. In our study, the least educated patients declared the most active attitude. This result can be interpreted in the context of the operationalization of ATH. Patients were asked about their knowledge, emotions, and treatment implementation plans in relation to the particular visit that took place. The least educated participants might feel they gained more than more educated ones because of the lower level of previsit activation.

### Limitations of the study

The present study has several limitations. First, the response rate of the facilities participating in the study was very low, which is typical for studies regarding health-care professionals [34]. Therefore, it is not entirely clear if the facilities participating in the study were not biased in some way and subsequently if the patients recruited were representative of the general population of older adults. Also, the conclusions must be limited to primary health-care patients. Accessing a primary care facility requires an older person to be independent and in relatively good health. Those who were not well, most likely even if they were able to attend a GP, did not give their consent to participate. Moreover, in investigating the ATH, we concentrated on patients’ experience rather than actual behavior. Further research should especially address the issue of the relationship between ATH and patients’ actions regarding health.

## Conclusions

The study allowed us to investigate a large group of primary-care patients; thus, we were able to separate different age subgroups within the studied population of older adults and study gender differences in the attitude toward treatment and health. The multidimensional assessment of ATH was also an asset.

Our results suggest that more, or maybe a different type of, effort is required to activate men. Gender differences tended to be most pronounced in a younger group of senior patients (aged 50–64) or among the oldest old (85+). Additionally, the perception of one’s own health as being very good has different consequences for men and women; it decreases involvement and the sense of responsibility in men, and increases activation in women. Interventions and programs addressing the issue of ATH shall therefore consider gender, age, and subjective health status as important, interrelated factors. Building a sense of efficacy and individual responsibility for health, providing information about the means of health promotion and disease prevention, and recognizing health-related expectations is recommended especially for men who feel well and are less advanced in age (50–64). In other words, this group needs to gain more knowledge of their health, together with a sense of control over it. Low levels of positive emotions also require intervention: Building men’s sense of self-efficacy might increase their optimism and hope. One way to achieve this during a doctor–patient encounter is to increase the men’s awareness and engagement through motivational interviewing techniques. Both training doctors to better handle patient engagement and training patients to feel more responsible and in control of their health can reinforce these processes. Another issue, which would extend the scope of the present article, but which is closely related to achieving these goals, would be discovering psychological factors (which may include, but are not limited to, specific cognitions about health-promoting and disease-preventing activities and their origins) related to low ATH in men. Also, other sociodemographic characteristics, such as SES, marital status, and education may be related to patients’ ATH, and therefore should be addressed.

In the clinical setting, the aforementioned groups of patients require a more careful initial diagnosis of a patient’s illness and health cognitions, emotions, and motivation, as well as deliberate and tailored interventions aimed at improving ATH. Further research should also address possible cultural differences in issues investigated in the present study.

## Additional file

Additional file 1: Table S1. Pairwise comparisons in significant interactions for ATH. The table presents results of pairwise comparisons for significant interactions of gender and investigated variables in all dimensions of ATH (DOCX 18 kb)

## Abbreviations

ATH: Attitude toward treatment and health
GENLIN: Generalized Linear Models
GP: general practitioner
PRACTA-ATH: PRACTA Attitude toward Treatment and Health Questionnaire
SAH: self-assessed health
SES: socio-economic status

## References

[1] World Health Organization. Good health adds life to years. Global brief for world health day 2012. 2012. http://www.who.int/ageing/publications/whd2012_global_brief/en/. Accessed 10 Feb 2016.

[2] Frosch DL, Rincon D, Ochoa S, Mangione CM (2010). Activating seniors to improve chronic disease care: results from a pilot intervention study. J Am Geriatr Soc.

[3] Begum N, Donald M, Ozolins IZ, Dower J (2011). Hospital admissions, emergency department utilization and patient activation for self-management among people with diabetes. Diabetes Res Clin Prac.

[4] Remmers C, Hibbard J, Mosen DM, Wagenfield M, Hoye RE, Jones C (2009). Is patient activation associated with future health outcomes and healthcare utilization among patients with diabetes?. J Ambul Care Manage.

[5] Thompson TL, Robinson JD, Beisecker AE: The older patient-physician interaction**.** *In* Handbook of communication and aging research. Edited by Nussbaum JF, Coupland J. Mahwah. NJ: Lawrence Erlbaum Associates; 2004:451–577.

[6] Davis MM, Bond LA, Howard A, Sarkisian CA (2011). Primary care clinician expectations regarding aging. Gerontologist.

[7] Jakubowska-Winecka A, Włodarczyk D, Jakubowska-Winecka A, Włodarczyk D (2007). Psychologiczne aspekty choroby i chorowania [psychological aspects of illness and being ill]. Psychologia w praktyce medycznej [psychology in medical practice].

[8] Sint Nicolaas SM, Schepers SA, van den Bergh EEM, Evers AWM, Hoogerbrugge PM, Grootenhuis MA, Verhaak CM (2016). Illness cognitions and family adjustment: psychometric properties of the illness cognition questionnaire for parents of a child with cancer. Support Care Cancer.

[9] Baron RA, Byrne D (1984). Social psychology understanding human interaction.

[10] van den Berg H, Mansted ASR, van der Plight J, Wigboldus DHJ (2006). The impact of affective and cognitive focus on attitude formation. J Exp Soc Psychol.

[11] Rosenberg MJ, Hovland CI, Rosenberg MJ, Hovland CI (1960). Cognitive, affective and behavioral components of attitudes. Attitude organization and change: an analysis of consistency among attitude components.

[12] Verhoof EJA, Maurice-Stam H, Heymans HSA, Evers AWM, Grootenhuis MA (2014). Psychosocial well-being in young adults with chronic illness since childhood: the role of illness cognitions. Child Adolesc Psychiatry Ment Health.

[13] Hudson JL, Bundy C, Coventry P (2016). What are the combined effects of negative emotions and illness cognitions on self-care in people with type 2 diabetes? A longitudinal structural equation model. Psychol Health.

[14] Hudson JL, Bundy C, Coventry PA (2014). Exploring the relationship between cognitive illness representations and poor emotional health and their combined association with diabetes self-care. A systematic review with meta-analysis. J Psychosom Res.

[15] Ek S (2015). Gender differences in health information behaviour: a Finnish population-based survey. Health Promot Int.

[16] Crimmins M, Kim JK, Solé-Auró A (2011). Gender differences in health: results from SHARE, ELSA and HRS. Eur J Pub Health.

[17] McLean CP, Anderson ER (2009). Brave men and timid women? A review of the gender differences in fear and anxiety. Clin Psychol Rev.

[18] Carayanni V, Stylianopoulou C, Koulierakis G, Babatsikou F, Koutis C (2012). Sex differences in depression among older adults: are older women more vulnerable than men in social risk factors? The case of open care centers for older people in Greece. Eur J Ageing.

[19] Hendriks M, Rademakers J: Relationships between patient activation, disease-specific knowledge and health outcomes among people with diabetes; a survey study**.** BMC Health Serv Res 2014,14: 393. doi:http://doi.org/10.1186/1472-6963-14-393. 10.1186/1472-6963-14-393 PMC4175625 25227734

[20] Penno G, Solini E, Bonora E, Fondelli C, Orsi E, Zerbini G (2013). Gender differences in cardiovascular disease risk factors, treatments and complications in patients with type 2 diabetes: the RIACE Italian multicentre study. J Intern Med.

[21] Hendriks SH, Hartog LC, Groenier KH, Maas AHEM, van Hateren KJJ, Kleefstra N, Bilo HJG. Patient activation in type 2 diabetes: does it differ between men and women? J Dibetes Res. 2016; doi:10.1155/2016/7386532. 10.1155/2016/7386532 PMC5021491 27656658

[22] Bos-Touwen I, Schuurmans M, Monninkjof EM (2015). Patient and disease characteristics associated with activation for self-management in patients with diabetes, chronic obstructive pulmonary disease, chronic heart failure and chronic renal disease: a cross-sectional survey study. PLoS One.

[23] Chubak J, Anderson KW, Saunders KW, Hubbard RA, Tuzzio L, Liss DT, Morales LS, Reid RJ (2012). Predictors of 1-year change in patient activation in oler adults with diabetes mellitus and heart disease. J Am Geriatr Soc.

[24] Gorman BK, Read JG (2006). Gender disparities in adult health: an examination of three measures of morbidity. J Health Soc Behav.

[25] World Health Organization. World report on disability. 2011 World Disability Report. http://www.who.int/disabilities/world_report/2011/en/. Accessed 4 Feb 2016.

[26] Smith SG, Curtis LM, Wardle J, vonWagner C, Wold MS (2013). Skill set or mind set? Associations between health literacy, patient activation and health. PLoS One.

[27] Mavaddat N, Kinmonth AL, Sanderson S (2011). What determines self-rated health (SRH)? A cross-sectional study of SF-36 health domains in the EPIC-Norfolk cohort. J Epidemiol Community Health.

[28] Greene J, Hibbard JH (2012). Why does patient activation matter? An examination of the relationships between patient activation and health-related outcomes. J Gen Intern Med.

[29] Blakemore A, Hann M, Howells K, Panagioti M, Sidaway M, Reeves D, Bower P: Patient activation in older people with long-term conditions and multimorbidity: correlates and change in a cohort study in the United Kingdom**.** BMC Health Serv Res 2016, 16:582–593. doi: http://doi.org/10.1186/s12913-016-1843-2. 10.1186/s12913-016-1843-2 PMC5069882 27756341

[30] Wlodarczyk D, Chylińska J, Lazarewicz M, Rzadkiewicz M, Jaworski M, Adamus M, et al. Enhancing doctors’ competencies in communication with and activation of older patients: the promoting active aging (PRACTA) computer-based intervention study. J Med Internet Res. 2017;19(2) 10.2196/jmir.6948 PMC5343213 28228370

[31] Dunteman GH, Moon-Ho RH (2006). An introduction to generalized linear models.

[32] Klemenc-Ketis Z, Krizmaric M, Kersnik J (2013). Age- and gender-specific prevalence of self-reported symptoms in adults. Centr Eur J Pub Health.

[33] Haslbeck JMR, Schaffer D (2012). Chronic illness self-management while living alone in later life: a systematic integrative review. Res Ageing.

[34] Groenwegen PP, Gres S, Schafer W. General practitioners’ participating in a large multicountry combined general practitioner-patient survey: recruitment procedures and participation rate. Int J Family Med. 2016. doi:10.1155/2016/4929432. 10.1155/2016/4929432 PMC4800081 27047689

