# Supplementary material for: The role of gender in the active attitude toward treatment and health among older patients in primary health care—self-assessed health status and sociodemographic factors as moderators
Source: BMC Geriatr. 2017 Dec 8;17:284. doi: 10.1186/s12877-017-0677-z (PMC5721697; doi:10.1186/s12877-017-0677-z)
Supplement: Additional file 1: Table S1. — Pairwise comparisons in significant interactions for ATH. The table presents results of pairwise comparisons for significant interactions of gender and investigated variables in all dimensions of ATH (DOCX 18 kb) [file 12877_2017_677_MOESM1_ESM.docx]

Additional file 1: Table S1 Pairwise comparisons in significant interaction effects of ATH

| Variable | | Women | | Men | | p |
| --- | --- | --- | --- | --- | --- | --- |
| **ATH Cognitive** | | | | | | |
| Gender x Age | | M | SD | M | SD |  |
|  | 50-64 | 5.42 | 0.07 | 4.97 | 0.08 | 0.000 |
|  | 65-74 | 5.65 | 0.07 | 5.33 | 0.07 | 0.002 |
|  | 75-84 | 5.40 | 0.07 | 5.38 | 0.09 | 0.828 |
|  | 85+ | 5.15 | 0.12 | 5.17 | 0.14 | 0.930 |
| Gender x Health | |  |  |  |  |  |
|  | Very good | 5.70 | 0.19 | 4.95 | 0.17 | 0.004 |
|  | Good | 5.45 | 0.07 | 5.27 | 0.07 | 0.055 |
|  | Average | 5.49 | 0.06 | 5.56 | 0.07 | 0.389 |
|  | Poor | 5.07 | 0.07 | 5.11 | 0.08 | 0.650 |
|  | Very poor | 5.32 | 0.18 | 5.18 | 0.20 | 0.585 |
| Variable | | Women | | Men | | p |
| **ATH Positive Emotions** | | | | | | |
| Gender x Age | |  |  |  |  |  |
|  | 50-64 | 5.65 | 0.07 | 5.15 | 0.07 | 0.000 |
|  | 65-74 | 5.71 | 0.06 | 5.45 | 0.07 | 0.006 |
|  | 75-84 | 5.44 | 0.07 | 5.40 | 0.08 | 0.695 |
|  | 85+ | 5.39 | 0.11 | 5.32 | 0.13 | 0.690 |
| Gender x Health | |  |  |  |  |  |
|  | Very good | 5.99 | 0.17 | 5.33 | 0.17 | 0.006 |
|  | Good | 5.57 | 0.06 | 5.52 | 0.07 | 0.631 |
|  | Average | 5.48 | 0.05 | 5.60 | 0.07 | 0.157 |
|  | Poor | 5.19 | 0.07 | 5.33 | 0.08 | 0.174 |
|  | Very poor | 5.52 | 0.16 | 4.88 | 0.17 | 0.007 |
| Variable | | Women | | Men | | p |
| **ATH Motivation** | | | | | | |
| Gender x Age | |  |  |  |  |  |
|  | 50-64 | 5.78 | 0.08 | 5.10 | 0.08 | 0.000 |
|  | 65-74 | 5.86 | 0.07 | 5.52 | 0.08 | 0.001 |
|  | 75-84 | 5.69 | 0.07 | 5.55 | 0.09 | 0.224 |
|  | 85+ | 5.52 | 0.13 | 5.39 | 0.14 | 0.477 |
| Gender x Health | |  |  |  |  |  |
|  | Very good | 6.07 | 0.19 | 4.88 | 0.16 | 0.000 |
|  | Good | 5.62 | 0.07 | 5.42 | 0.08 | 0.062 |
|  | Average | 5.65 | 0.06 | 5.72 | 0.07 | 0.499 |
|  | Poor | 5.47 | 0.08 | 5.46 | 0.09 | 0.949 |
|  | Very poor | 5.75 | 0.19 | 5.46 | 0.20 | 0.295 |
